# Analysis of Arabidopsis TPK2 and KCO3 reveals structural properties required for K^+^ channel function

**DOI:** 10.1080/19336950.2020.1825894

**Published:** 2020-10-04

**Authors:** Chihiro Uehara, Kota Takeda, Tatsuki Ibuki, Tadaomi Furuta, Naomi Hoshi, Ellen Tanudjaja, Nobuyuki Uozumi

**Affiliations:** a Department of Biomolecular Engineering, Graduate School of Engineering, Tohoku University, Sendai, Japan; b Bioscience and Biotechnology Center, Nagoya University, Nagoya, Japan; c School of Life Science and Technology, Tokyo Institute of Technology, Yokohama, Japan

**Keywords:** channel, potassium, two-pore, calcium, EF-hand, Arabidopsis

## Abstract

Arabidopsis thaliana contains five tandem-pore domain potassium channels, TPK1-TPK5 and the related one-pore domain potassium channel, KCO3. Although KCO3 is unlikely to be an active channel, it still has a physiological role in plant cells. TPK2 is most similar to KCO3 and both are localized to the tonoplast. However, their function remains poorly understood. Here, taking advantage of the similarities between TPK2 and KCO3, we evaluated Ca^2+^ binding to the EF hands in TPK2, and the elements of KCO3 required for K^+^ channel activity. Presence of both EF-hand motifs in TPK2 resulted in Ca^2+^ binding, but EF1 or EF2 alone failed to interact with Ca^2+^. The EF hands were not required for K^+^ transport activity. EF1 contains two cysteines separated by two amino acids. Replacement of both cysteines with serines in TPK2 increased Ca^2+^ binding. We generated a two-pore domain chimeric K^+^ channel by replacing the missing pore region in KCO3 with a pore domain of TPK2. Alternatively, we generated two versions of simple one-pore domain K^+^ channels by removal of an extra region from KCO3. The chimera and one of the simple one-pore variants were functional channels. This strongly suggests that *KCO3* is not a pseudogene and KCO3 retains components required for the formation of a functional K^+^ channel and oligomerization. Our results contribute to our understanding of the structural properties required for K^+^ channel activity.

## Introduction

Potassium is a major cation in plant cells and it contributes to intracellular osmotic pressure and membrane potential formation [1]. To supply K^+^ to various types of cells, plants have evolved K^+^ channels as well as two classes of K^+^ transporters, Trk/Ktr/HKT and KT/HAK/KUP [2]. K^+^ channels and Trk/Ktr/HKT-type K^+^ transporters share structural similarities in their K^+^ conducting pore formed by a membrane-pore-membrane structure, that differentiates them from KT/HAK/KUP-type K^+^ transporters [3]. The Arabidopsis genome encodes nine six-membrane-spanning K^+^ channels with voltage sensors and six K^+^ channels without voltage sensors. The latter comprises the one-pore Kir-like channel, KCO3 and five tandem-pore domain K^+^ channels, TPK1-TPK5 [4,5]. While the voltage-dependent K^+^ channels have been well studied, much less is known about KCO3 and some of the tandem-pore domain K^+^ channels [6]. KCO3 and TPK1-TPK5 share relatively high homology but KCO3 lacks the first pore domain and the second transmembrane span of the TPKs. KCO3 and the TPKs with exception to TPK4 have tandem EF-hand motifs in their cytosolic C-terminal domains. These motifs are generally considered to function as Ca^2+^-binding domains. Unlike the TPKs, KCO3 does not appear to have an ion transport activity. However, *KCO3* is expressed in the vascular tissue throughout the plant, and KCO3 forms homodimers and localizes to the tonoplast, suggesting some biological function of KCO3 *in vivo* [5,7]. Under high osmolarity conditions, an Arabidopsis *kco3* mutant has shorter roots than the wild type [7]. *KCO3* is expressed during seed development and its expression is altered by auxin treatment [8].

In contrast to KCO3, TPK1-TPK5 are functional channels, which was confirmed by patch clamp recordings and functional complementation tests in *E. coli* [9–16]. They likely function as dimers [6]. TPK4 localizes to the plasma membrane while the other four TPKs, TPK1, 2, 3 and 5 reside in the tonoplast. The biological role of TPK1 and TPK4 has been characterized, but less is known about the function of TPK2, TPK3 and TPK5. Calcium ions (Ca^2+^) play an important role as a ubiquitous second messenger in numerous biological processes in cells and are involved in the regulation of many ion transport systems. An extracellular concentration of 30 mM Ca^2+^ inhibits TPK4 function [9]. Patch clamp recording of the TPK1 homolog from tobacco, NtTPK1, shows that excluding Ca^2+^ from the cytosolic side enhances the K^+^ current [17]. TPK1 represents the voltage-independent potassium-selective vacuolar K^+^ (VK) channel identified by electro-physiological measurements [13,14]. Detailed studies of TPK1 activity revealed that 14-3-3 proteins, pH and Ca^2+^ function in the regulation of TPKs. The EF hands in TPK1 interact with Ca^2+^. Phosphorylation mediated by Ca^2+^-dependent protein kinases like CPK3 promotes interaction of a 14-3-3 protein (GRF6) with the cytosolic N-terminal domain of TPK1 during salt-stress adaptation [11,12]. Ca^2+^ enhances TPK transport activity, but it is unclear whether interaction of the EF-hands with Ca^2+^ is the direct cause of this enhancement [11]. Moreover, TPK4 is lacking EF-hands, even though its transport activity is affected by Ca^2+^ [9]. The overall importance of Ca^2+^ binding to the EF-hands for TPK function therefore remains elusive.

Due to the low transcript abundance of *TPK2* and the fact that KCO3 does not appear to have channel activity, little is known about the function of both [5,7]. TPK2 and KCO3 share high similarity and they localize to the tonoplast. Taking advantage of the similarities between TPK2 and KCO3, we characterized the function of the EF-hands in TPK2 and explored the structures required to allow KCO3 to function as a K^+^ channel. Specifically, we examined the Ca^2+^ binding properties of TPK2 and whether TPK2 function was dependent on Ca^2+^. Based on the fact that TPK2 was functional when expressed in *E. coli* we generated a chimeric protein of KCO3 and TPK2, as well as monomeric KCO3 variants, and evaluated their function. Our results suggest that KCO3 is not an active K^+^-channel because it lacks the intermediate region found in TPK-type K^+^ channels.

## Results

### Requirement of the EF-hands for TPK2-mediated K^+^ channel activity

**Figure 1.**
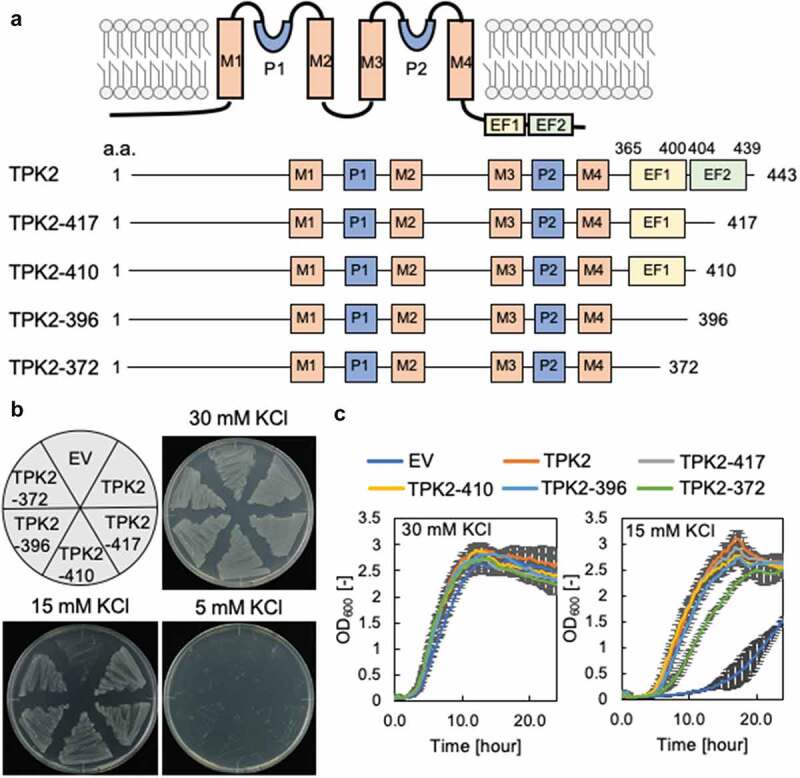
Contribution of EF-hand domains to K^+^ transport activity of TPK2. (a) Structure model of TPK2 and truncated variants. TPK2 has four transmembrane domains (M1-4), two-pore domains (P1-2) and two predicted EF-hands, EF-1 (L365-K400) and EF-2 (K404-T439). TPK2-417 contains EF-1 and a part of EF-2. TPK2-410 contains EF-1. TPK1-396 contains a part of EF-2. TPK1-372 contains a part of EF-2. (b) and (c) Growth of *E. coli* LB2003 expressing TPK2 and its truncated variants on solid medium (B) and in liquid medium (C). The error bars represent mean ± S.D. (n = 3). EV = empty vector.

TPK2 contains tandem EF-hand sequences, EF1 and EF2, in its C-terminal region. To assess the role of these EF-hands for TPK2-mediated K^+^ channel activity, we performed complementation assays of *E. coli* LB2003, a strain that is unable to grow at low K^+^ concentrations unless a functional K^+^ transport system is present [18,19]. *E. coli*, instead of eukaryotic cells like yeasts, is a suitable heterologous expression system to determine the activity of organellar transporters like the tonoplast-localized TPK2 [19] because they can be expected to be inserted into the plasma membrane. LB2003 transformed with either full-length TPK2 or with C-terminally truncated forms of TPK2 lacking EF2 or both of the EF hands (Figure 1(a)) was grown under K^+^-limiting conditions (15 mM KCl). The cells containing full-length TPK2 grew well on solid medium containing 15 mM KCl, consistent with previous results [10]. Interestingly, LB2003 containing the truncated forms of TPK2 also was able to grow under the same conditions. Cells containing the Arabidopsis inward-rectifying K^+^ channel KAT1 as a positive control grew as well, but cells containing the empty vector did not grow (Figure 1(b)). In liquid culture, the variants without EF2 (TPK2-417 and TPK2-410) and without both EF1 and EF2 (TPK2-396 and TPK2-372) grew in medium containing 15 mM KCl, although the cells expressing TPK2-372 grew somewhat more slowly (Figure 1(c)). These results indicate that EF1 and EF2 are not required for TPK2-mediated K^+^ channel activity.

### Binding of Ca^2+^ to EF1-EF2

**Figure 2.**
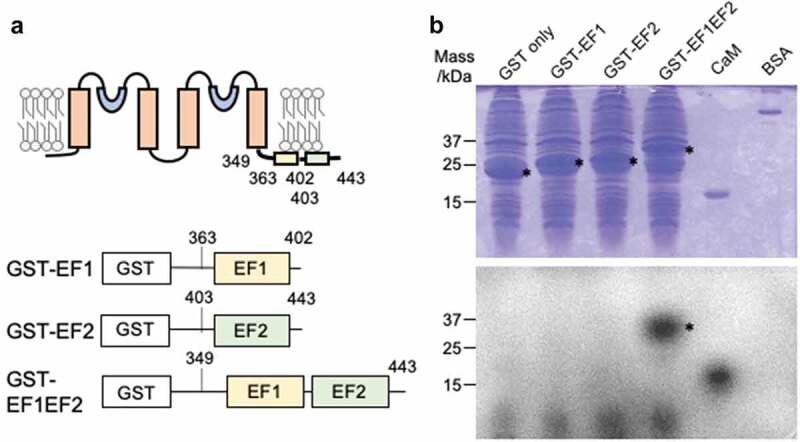
Ca-binding to the EF-hands of TPK2. (a) Schematic depicting constructs for expression of GST-fused EF1, EF2 and C-terminal region of TPK2 including both EF-1 and EF-2. (b) SDS-PAGE of GST-fusion proteins stained with coomassie brilliant blue (top) and ^45^Ca-binding assay of TPK2 EF-hand performed on the same gel (bottom). * marks bands corresponding to TPK2-polypeptides containing EF hands.

To assess the function of EF1 and EF2 in TPK2, we conducted a Ca^2+^ binding test with EF1 and EF2 (Figure 2(a)). Fusion proteins consisting of either the entire C-terminal cytosolic domain containing both EF1 and EF2, or EF1 and EF2 individually attached to the C-terminus of GST were expressed in *E. coli* (Figure 2(b)). The fusion proteins were subjected to a ^45^Ca-binding assay (Figure 2(b)). Calmodulin (CaM) and BSA were used as positive and negative controls, respectively. EF1 or EF2 alone did not bind ^45^Ca, but interaction of ^45^Ca with the entire C-terminal cytosolic domain of AtTPK2 was observed. Hence, Ca^2+^ was only able to bind to the protein containing both EF1 and EF2.

### Role of Cys in EF2 of TPK2

**Figure 3.**
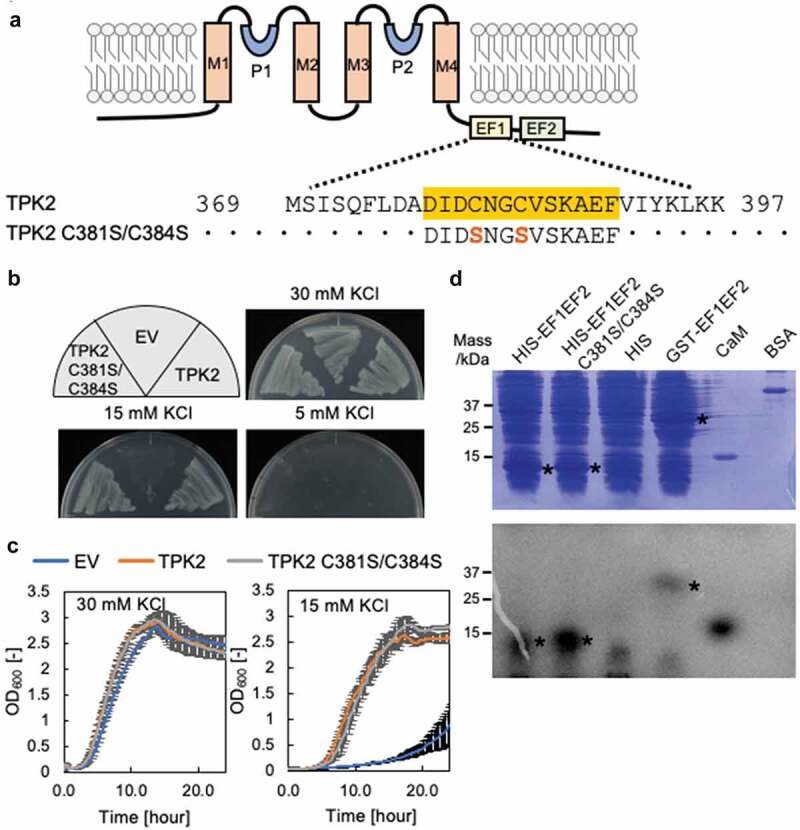
Role of dual Cys in EF-1 for Ca^2+^ binding of TPK2. (a) Schematic showing part of the amino acid sequence of EF1 with C381 and C384 and the corresponding sequence in the C381S/C384S variant. (b) Growth of *E. coli* LB2003 expressing the C381S/C384S variant on solid medium (B) and in liquid medium (c). The error bars represent mean ± S.D. (n = 3). (d) SDS-PAGE of His-tag fusion proteins stained with Coomassie Brilliant Blue (top) and ^45^Ca-binding assay performed on the same gel (bottom). * marks bands corresponding to TPK2-polypeptides containing EF hands. EV is empty vector.

**Figure 4.**
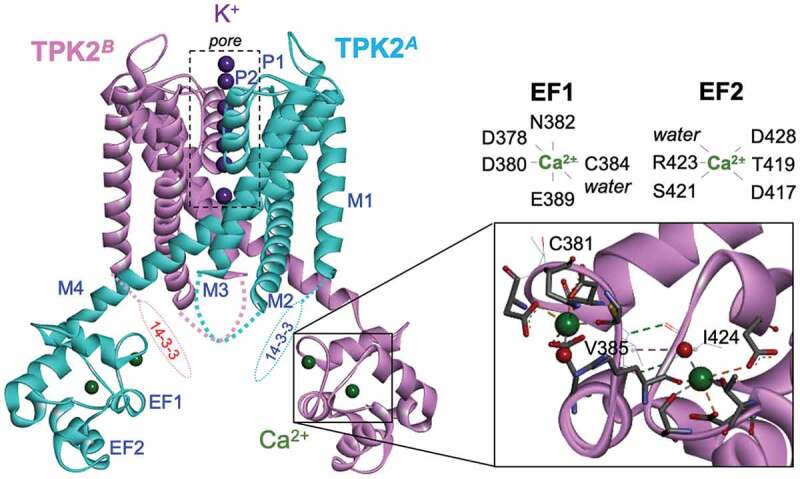
Structural model of AtTPK2. Open state structure of a TPK2 dimer comprising the structural elements [14-3-3]-M1-P1-M2-M3-P2-M4-EF1-EF2. The detail shows a close-up view of the EF-hand regions (EF1 and EF2). Proteins are represented by ribbons (subunits A and B are shown in cyan and pink, respectively). Ions (K^+^ [pore], Ca^2+^ [EF]) and coordinated water molecules [EF] are represented by spheres in purple, green, and red, respectively. Coordinated residues at EF1 and EF2 sites are represented by sticks. The hydrogen bonding residues (V385 and I424) and non-coordinated Cys residue (C381) are represented by lines.

EF1 in TPK2 contains two Cys separated by two amino acids (Figure 3(a)), a unique feature that is not present in the EF hands of TPK1, 3, 4 and 5, KCO3, the TPK homologs. Some calmodulin homologs possess a single Cys that corresponds to C384 in TPK2, but lack the second Cys corresponding to C381 [20–22]. The conserved Cys participates in the interaction with Ca^2+^ in calmodulin [21]. We hypothesized that the two adjacent cysteine residues in TPK2 may form disulfide bonds, leading to a conformational change of the EF hand and resulting in regulation of Ca^2+^ binding ability. To examine the role of Cys in TPK2, we replaced both Cys in EF1 with Ser (Figure 3(a)) and expressed the resulting TPK2-C381S/C384S variant in *E. coli* strain LB2003. The growth rate of TPK2-C381S/C384S-expressing *E. coli* in media containing low concentrations of KCl was similar to that of the cells expressing wild-type TPK2 (Figure 3(b,c)). However, ^45^Ca-binding assays with the EF1-EF2 peptides showed that conversion of Cys to Ser in C381S-C384S increased the affinity for Ca^2+^ compared to the wild type (Figure 3(d)). To investigate the cause of this affinity increase, we conducted structural modeling of TPK2 (Figure 4 and Supplemental Figure S1). The Ca^2+^ binding motif of EF1 consists of amino acids 378 to 389 with the sequence DIDCNGCVSKAE (amino acids involved in Ca^2+^ binding are underlined). Specifically, the bound Ca^2+^ is coordinated to the four side chains of D378, D380, N382 and E389, one main chain of C384 and one H_2_O molecule. Ca^2+^ in EF2 is also hexa-coordinated to the four side chains of D417, T419, S421 and D428, one main chain of R423 and water (417-DRTNSGRITLLD-428). The distance between C381 and C384 was large in the model structure (Figure 4); thus, they might not be a pair forming a disulfide bond. The conversion of C384 to Ser (C381S-C384S variant) resulted in the introduction of strong polarity of the side chain at position 384. This side chain might then be able to interact with the side chain of N382 that binds to Ca^2+^, further enhancing Ca^2+^ interaction.

### Insertion of a region from TPK2 into KCO3 confers K^+^ channel activity

**Figure 5.**
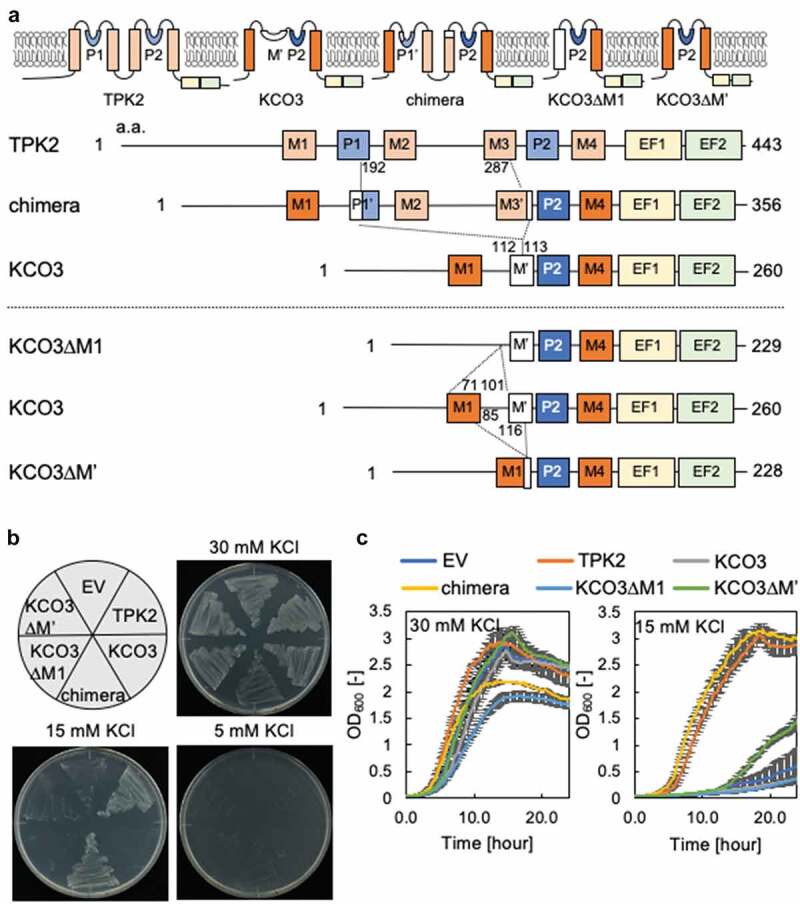
K^+^ transport activity of KCO3-TPK2 chimera. (a) Schematic diagram of the protein chimera between KCO3 and TPK2 and of KCO3 variants with deleted domains. The chimeric two-pore domain channel was generated by insertion of the missing part of the two-pore domains from TPK2 into KCO3. KCO3∆M1 lacks residues L71 to A101. KCO3∆M’ lacks residues from Y85 to I116. (b and c) Growth of *E. coli* LB2003 expressing the KCO3/ TPK2 chimera or variants of KCO3 on solid medium (b) and in liquid medium (c). The error bars represent mean ± S.D. (n = 3). EV = empty vector.

**Figure 6.**
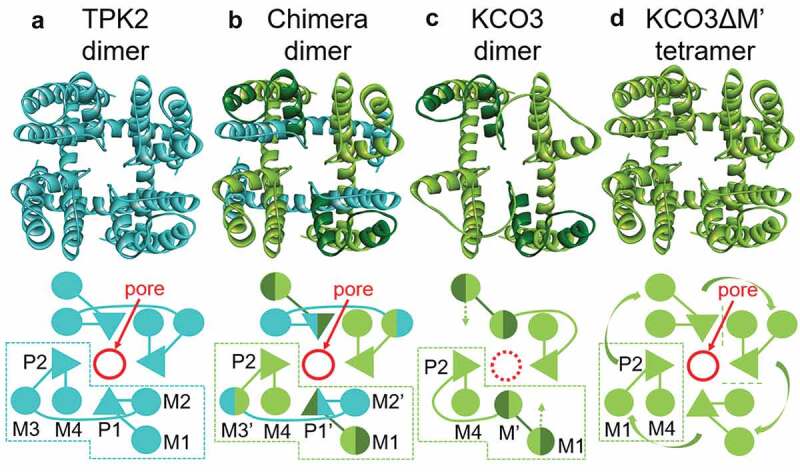
Top-view topologies of the transmembrane regions of TPK2, chimera, KCO3, and KCO3ΔM’. The structural models (top) and schematic pictures (bottom) of TPK2, chimera, KCO3 and KCO3ΔM’ are shown in (ad), respectively. The TPK2 and KCO3 originated residues are shown in cyan and green, respectively (where the M’ region of KCO3 are in dark green for indicating the deletion region). In (D), the M1 helix of KCO3ΔM’ would be rearranged by the deletion of the M’ region, and might move to the M3 position of TPK2 (indicated by green curved arrows). Moreover, in (c), the M1 helix of KCO3 might move inside toward a M2-like position of TPK2 (indicated by dashed green arrows), taking a more packed conformation. The depicted dimerization of KCO3 is based on the data in Rocchetti et al. [7]. The model structures of (bD) were manually created based on structural insights gained from the TPK2 topology.

The closest homolog to KCO3 within the TPK family is TPK2. KCO3 does not have K^+^ channel activity because it is lacking the intermediate region which contains the first GYG sequence, a consensus sequence of the pore domain in TPKs [6]. Nevertheless, KCO3 has been reported to have physiological functions *in vivo* [5,7]; however, the exact nature of these functions is largely unknown. We speculated that KCO3 could gain K^+^ transport activity by transferring this missing region from TPK2 to KCO3. We therefore constructed a KCO3-TPK2 chimera (Figure 5(a)) and tested K^+^ uptake activity of this variant (Figure 5(b,c)). The KCO3-TPK2 chimera complemented the K^+^ uptake deficiency of *E. coli* strain LB2003 on solid medium and in liquid culture. This suggested that the pore region of KCO3 is able to function as a K^+^ channel. To further investigate the potential KCO3 activity, we designed two truncated forms of KCO3, KCO3ΔM1 and KCO3ΔM’ by removing the M1 or M’ helices from KCO3 and converting it to a simple one-pore K^+^ channel structure. In growth assays, KCO3ΔM1 was unable to complement *E. coli* strain LB 2003 (Figure 5(b,c)). In contrast, the growth rate of LB2003 containing KCO3ΔM’ in liquid medium was higher than that of cells containing KCO3ΔM1 or the empty vector. These results suggest that KCO3 was able to form oligomers [7] (Figure 6).

## Discussion

Our study provides experimental evidence for Ca^2+^ binding to the tandemly arranged two EF-hands of TPK2, and for the presence of structural elements required for channel activity in KCO3. This suggests that KCO3 might have originated from a functional tandem-pore domain K^+^ channel such as TPK2, possibly by a deletion event that removed part of the genomic sequence of a *TPK*.

Although our study showed that the EF-hands in TPK2 serve as Ca^2+^ binding sites (Figure 2), removing the C-terminal region containing the EF-hands did not affect K^+^ transport activity of TPK2 (Figure 1). Intracellular Ca^2+^ is known to be involved in the regulation of plant TPK-type channels [9,11,12] but the exact mechanism of this regulation is unclear. The EF-hand motif is well conserved within the TPK family but the significance of Ca^2+^ binding to EF-hands for transport activity has not yet been determined. In tobacco TPK1, which possesses two EF-hands in tandem in its C-terminal region, intracellular Ca^2+^ significantly increased transport activity [17], but the cause of this effect remains to be investigated. TPK1 in Arabidopsis has two EF-hands like TPK2, which can bind Ca^2+^ [11]. However, Ca^2+^ is also required for activation of Ca^2+^-dependent kinase which promotes the binding of regulatory 14-3-3 protein to the N-terminal region of TPK1 [11,12]. Therefore, it is not yet clear whether interaction between intracellular Ca^2+^ and EF-hands plays a major role in the regulation of TPK1. Moreover, TPK4 has no EF-hands, and thereby its Ca^2+^-dependent regulation does not involve EF-hands [9].

Our Ca^2+^-binding assay showed that Ca^2+^ binding occurred when the entire C-terminal region containing both EF1 and EF2 was present but no Ca^2+^ binding was found with a single EF1 or EF2 (Figure 2). EF1 and EF2 depended on each other to form the correct structure. Our molecular model indicates that EF1 and EF2 are stabilized by two hydrogen bonds of NH-CO in the polypeptide chain of V385 of EF1 and I424 of EF2 and the hydrophobic bond formed by the side chains of the residues and surrounding hydrophobic clusters (Figure 4). Cys often serves as a sensing amino acid for the intracellular redox state. However, the structural model shows that the side chains of both Cys381 and Cys384 extend in opposite direction to Ca^2+^, and thereby it is unlikely that a disulfide bond can be formed between Cys381 and Cyc384. This is supported by the finding that substitution of Ser for Cys384 increased Ca^2+^ binding to EF1 (Figure 3(d)). Arabidopsis calmodulin 7 contains a single Cys in its second EF hand, which corresponds to C384 in TPK2. The oxygen of the peptide chain of Cys forms a coordinated bond with Ca^2+^ [21]. A similar interaction of the free Cys is also observed in guanylyl cyclase activator protein 1 belonging to the calmodulin superfamily [22]. Both EF1 and EF2 in TPK2 are almost identical to the proposed canonical EF-hand motif, DxDxDG [23].

Patch clamp-recording of whole vacuoles isolated from KCO3-overexpressing Arabidopsis led to the conclusion that KCO3 is not a functional K^+^ channel [7]. On the other hand, visualization of KCO3 assembly using GFP-KCO3 in Arabidopsis revealed that KCO3 forms stable homo-dimers in leaves but not monomers or tetramers [7]. The minimal KCO3 (KCO3M’) variant and the KCO3-TPK2 chimera engineered for this study revealed which fundamental structures are required for K^+^ channel function (Figures 5 and 6). Because four K^+^ channel subunits assemble to form a K^+^ conductive pore, KCO3ΔM’ may be expected to form a tetramer as well. The ability of KCO3M’ or the KCO3-TPK2 chimera to complement the growth defect of *E. coli* LB2003 indicates that the first TM, the last TM and the pore region in KCO3 are able to assemble as a tetramer to form a functional K^+^ channel. KCO3M’ is therefore similar to one-pore Kir channels in animal cells [24]. An Arabidopsis null mutant of *kco3* shows reduced root growth, which can be complemented by reintroduction of KCO3 [7]. This likely physiological function of KCO3 and the ability of KCO3 to assemble as a tetramer point to a potential role of KCO3 as a modulator of other K^+^ channels. This is reminiscent of the silent K^+^ channel AtKC1, a member of the family of voltage-dependent K^+^ channels [25]. AtKC1 functions as a modulator of K^+^ channels in plant cells [26]. These nonfunctional channel homologs may act as negative regulators in plant cells.

## Materials and methods

### Strains and plasmids

*E. coli* strain BL21 was used as host for protein expression. All primers used in this study are listed in Supplementary Table 1. The coding sequence of AtTPK2 was amplified by polymerase chain reaction (PCR) using a pair of primers, KCO2-BglII-SacI-sta and KCO2-stop-Bam-R (Table S1). The PCR product was digested with *Sac*I and *BamH*I and inserted into the *Sac*I and *BamH*I sites in pPAB404. For the serial C-terminal deletion constructs of TPK2, pPAB404-AtTPK2-372, 396, 410 and 417, a set of primers with KCO2-BglII-SacI-sta as the forward primer, and one of the corresponding reverse primers listed in Table S1 was used for PCR amplification. The PCR products were inserted into the *Sac*I and *BamH*I sites in pPAB404.

For construction of pPAB404-AtTPK2C381S/C384, a two-step PCR protocol was used. In the first step, PCR was performed with pPAB404-AtTPK2 as a template using a pair of primers, C381S+C384S sense and pPAB-150bpReverse (Table S1). The resulting PCR product was used as a reverse primer in a second PCR reaction to amplify the entire sequence of TPK2 using pPAB-120bpForward as a forward primer (Table S1). The PCR product was digested with *Sac*I and *BamH*I and inserted into the same sites of pPAB404. The coding sequence of AtKCO3 was amplified by PCR with a pair of primers, KCO3-Start and KCO3-End (Table S1). The PCR product was inserted into *Sac*I and *BamH*I of pPAB404. The constructs encoding a chimera of TPK2 and KCO3 (chimera) or versions of KCO3 with deleted domains (KCO3∆M1, KCO3∆M’) were generated by two-step PCR with the primers listed in Table S1, and the PCR products were inserted into *Sac*I and *BamH*I of pPAB404.

For GST-fused protein expression in *E. coli*, pGEX-EF1EF2 (consisting of amino acid 349 to the C-terminus of TPK2), pGEX-EF1 (consisting of amino acids 363 to 402 of TPK2) and pGEX-EF2 (consisting of amino acids 403 to the C-terminus of TPK2) the corresponding sequences were inserted between *BamH*I and *EcoR*I in pGEX-T. For His tag-fused protein expression in *E. coli*, pET-EF1EF2 (consisting of amino acid 349 to the C-terminus of TPK2) and pET-EF1EF2-C381S/C384S (consisting of amino acid 349 to the C-terminus of TPK2) the corresponding sequences were inserted between *Nde*I and *BamH*I in pET-16b.

### Growth tests

Transformants of *E. coli* strain LB2003 (F-, *thi, lacz, gal, rha*, ∆*kdpFABC5, trkD1*, ∆*trkA*) were grown in minimal medium (46 mM Na_2_HPO_4_, 23 mM NaH_2_PO_4_, 8 mM (NH_4_)_2_SO_4_, 0.4 mM MgSO_4_, 6 mM FeSO_4_, 10 μg/ml thiamine and 1% glucose with or without 1% agarose) supplemented with 25 µg/mL Na-ampicillin and 30 mM KCl at 30 °C for 16 h [27,28]. For liquid cultures, cells were grown to approximately OD_600_ = 0.5 and then harvested by centrifugation (15,000 rpm, 1 min). Cells were washed with sterilized water twice, and then re-suspended with 500 µL of minimal medium supplemented with 25 µg/mL Na-ampicillin, 0.5 mM IPTG and 15 or 30 mM KCl. Aliquots of 150 µL were transferred to 96-well microtiter plates and the cultures were incubated at 30 °C for 24 h. The growth of each strain was determined by measuring the OD_600_ every 15 min. Incubation and measurement were performed using a Synergy H1 microplate reader (BioTek). For growth test on solid medium, pre-cultured cells were streaked out on minimal medium supplemented with 1% agarose, 25 µg/mL Na-ampicillin, 0.5 mM IPTG and 5 mM, 15 mM or 30 mM KCl and then incubated at 30 °C for 24 h.

### ^45^Ca-binding assay

*E. coli* strains BL21 containing pGEX-EF1EF2, pGEX-EF1, pGEX-EF2, pET-EF1EF2 or pET-EF1EF2-C381S/C384S were grown in 2xYT medium (1.6% tryptone, 1% yeast extract and 0.5% NaCl) at 30 °C. Then, 4% of the pre-culture was transferred to fresh 2xYT medium and incubated until the OD_660_ reached 0.5. After addition of 0.2 mM Isopropyl β-D-1-thiogalactopyranoside, cultures were incubated at 30 °C for 3 h and then the cells were collected by centrifugation. The cells were resuspended with SDS-poly-acrylamide electrophoresis (SDS-PAGE) sample buffer and heated for 5 min. Samples were centrifuged to remove the debris, and the supernatants were subjected to SDS-PAGE (10% polyacrylamide). The gel was stained with Coomassie Brilliant Blue. For the Ca^2+^-binding assay, the gel was rinsed three times with buffer (10 mM 2-(N-morpholino) ethanesulfonic acid (MES)-KOH, pH 6.5) for 20 min each. Then, the gel was incubated with 1.8 MBq ^45^Ca in binding buffer (60 mM KCl, 5 mM MgCl_2_, MES-KOH, pH6.8) for 1 h with gentle shaking. Afterward, the gel was washed three times with binding buffer for 20 min each, dried, exposed to an imaging plate (FUJIFILM) and analyzed with a BAS-3000 phosphor imaging system (FUJIFILM).

### Structural modeling of TPK2

A model structure of TPK2 was created by homology modeling (Discovery Studio 2017 R2) using K^+^ channel (KcsA) [PDBid: 5VK6] as a template for the transmembrane domain (M1-P1-M2-M3-P2-M4) and guanylate cyclase-activating protein 1 (GCAP1) [PDBid: 2R2I] as a template for the EF-hand domain (EF1-EF2. The N-terminal 14-3-3 domain was not modeled. The sequence alignment for the structural modeling was obtained separately (https://www.ebi.ac.uk/Tools/msa/clustalo/) for each domain using Clustal Omega. The alignments were then combined and manually modified based on structural inspection taking helix continuity into consideration. When selecting the templates described above, no appropriate template sequence for the entire two-pore transmembrane domain was found by homology search (Blast); therefore, two subunits of a typical one-pore domain K^+^ channel were used. For modeling the EF-hand domain, only a few templates with weak similarity (E-value: 0.004–0.22) were found. One template (GCAP1) containing a single Cys corresponding to C384 in TPK2 was selected among those. Model structures of the chimera dimer, the KCO3 dimer, and the KCO3ΔM’ tetramer were manually created based on structural insights gained from the topology of a TPK2 dimer.

## Supplementary Material

Supplemental Material
