# Supplementary material for: Analysis of Arabidopsis TPK2 and KCO3 reveals structural properties required for K+ channel function
Source: Channels (Austin). 2020 Oct 4;14(1):336–46. doi: 10.1080/19336950.2020.1825894 (PMC7757853; doi:10.1080/19336950.2020.1825894)
Supplement: Supplemental Material [file KCHL_A_1825894_SM6536.pdf]

```
TPK2       1 MANDGNGDNNDDPLRQYLMNPRINPPPPSLLTLPENNDVTIPMPITPLELKNRLIFGSFV  60
                                                                       14-3-3
TPK2      61 RSRKESSLPIDALSQNPSTSSSATTSFSDSTDLLLPLTEPNKPVRKSKPTINFHRSKTAP 120
                                                 M1
TPK2     121 AMAAINNISHPNDPKTDQQSDSKTIVNQAVALLVVYLSLGVLIYWLNRDSYNVKQTHPVV 180
KcsA(1)   26 ----------------------WRCAGAATVLLVIVLLAGSYLAVLAERGAPGAQLITYP  63
                       P1                            M2
TPK2     181 DALYFCIVTMCTIGYGDITPDSVVTKLFSIFFVLVGFGFMDILLSGMVTYVLDLQENYML 240
KcsA(1)   64 RALWWSVATATTVGYGDLYPVTLWGRCVAVVVMVAGITSFGLVTAALATWFVGQCQQQ-- 121
                                                                M3
TPK2     241 ETARNESLNLNDRDKVRSYIIDVKKGRMRIRLKVGLALGVVVLCLGFGVLIMHFVE---- 296
KcsA(2)   26 ------------------------------WRCAGAATVLLVIVLLAGSYLAVLAERGAP  55
                               P2                               M4
TPK2     297 ---KIGWLDSFYFSVMSVTTVGYGDRAFNTLAGRLLAAMWLLVSTLAVARAILFLAESRV 353
KcsA(2)   56 GAQLITYPRALWWSVATATTVGYGDLYPVTLWGRCVAVVVMVAGITSFGLVTAALATWFV 115
                                        EF1 (Ca2+-binding)
TPK2     354 DKRNRERA-KKVL-GE--SMSISQFLDADIDCNGCVSKAEFVIYK--LKKMDKITE--KD 405
KcsA(2)  116 GQCQQQ------------------------------------------------------ 121
GCAP1     71 DFMEYVAALSLVLKGKVDQKLRWYFKLYDVDGNGCIDRGELLNIIKAIRAINRCNEAMTA 130
                     EF2 (Ca2+-binding)
TPK2     406 INPIGFQFDKLDRTNSGRITLLDLLESSTKDLPTATSI 443
GCAP1    131 EEFTNMVFDKIDINGDGELSLEEFMEGVQKDEVLLDIL 368
```

Supplemental Figure S1. Sequence alignment for the structural modeling of TPK2
The sequence alignments for each template (the potassium channel KcsA subunits (1) and (2) and the guanylate cyclase activating protein GCAP1) were obtained first using the Clustal Omega, then the alignments were arranged, where the light color regions for KscA (2) were manually modified. The template sequences of KcsA (1 and 2) and GCAP1 above are based on the PBD structures: 5VK6 and 2R2I, respectively. The N-terminal 14-3-3 regions (1-142) and internal loops (239-270) were not considered for the modeling.

```
                                   T192
TPK2      181  DALYFCIVTMCTIGYGDITPDSVVTKLFSIFFVLVGFGFMDILLSGMVTYVLDLQENYML  240
Chimera   102  VALYFFVVTFCTIGYGDITPDSVVTKLFSIFFVLVGFGFMDILLSGMVTYVLDLQENYML  161
KCO3      102  VALYFFVVTFC-------------------------------------------------  112
```

```
TPK2        1  MANDGNGDNNDDPLRQYLMNPRINPPPPSLLTLPENNDVTIPMPITPLELKNRLIFGSFV   60
Chimera     1  ------------------------------------------MPMTPSEFKNRLLFGSLP   18
KCO3        1  ------------------------------------------MPMTPSEFKNRLLFGSLP   18

TPK2       61  RSRKESSLPIDALSQNPSTSSSATTSFSDSTDLLLPLTEPNKPVRKSKPTINFHRSKTAP  120
Chimera    19  RSSSDPT---D-----------------------LQFTEPNVPPSLF--SLPEHNDDTAT   50
KCO3       19  RSSSDPT---D-----------------------LQFTEPNVPPSLF--SLPEHNDDTAT   50

TPK2      121  AMAAINNISHPNDPKTDQQSDSKTIVNQAVALLVVYLSLGVLIYWLNRDSYNVKQTHPVV  180
Chimera    51  DM---------APDQETEQSVSKSIARQALALLVVYLSLGVLIYWLTLDSDNAYQTHPVA  101
KCO3       51  DM---------APDQETEQSVSKSIARQALALLVVYLSLGVLIYWLTLDSDNAYQTHPVA  101
                           T192
TPK2      181  DALYFCIVTMCTIGYGDITPDSVVTKLFSIFFVLVGFGFMDILLSGMVTYVLDLQENYML  240
Chimera   102  VALYFFVVTFCTIGYGDITPDSVVTKLFSIFFVLVGFGFMDILLSGMVTYVLDLQENYML  161
KCO3      102  VALYFFVVTFC-------------------------------------------------  112
                          C112                                  F287
TPK2      241  ETARNESLNLNDRDKVRSYIIDVKKGRMRIRLKVGLALGVVVLCLGFGVLIMHFVEKIGW  300
Chimera   162  ETARNESLNLNDRDKVRSYIIDVKKGRMRIRLKVGLALGVVVLCLGFGFLIVHFVVKIGW  221
KCO3      113  -----------------------------------------------GFLIVHFVVKIGW  125
                                                                G113
TPK2      301  LDSFYFSVMSVTTVGYGDRAFNTLAGRLLAAMWLLVSTLAVARAILFLAESRVDKRNRER  360
Chimera   222  LDSFCFSVMMVTTVGFGDRAFNTWLGTFLAAVWLLVSTLAVARAFLFLADARADKRNRER  281
KCO3      126  LDSFCFSVMMVTTVGFGDRAFNTWLGTFLAAVWLLVSTLAVARAFLFLADARADKRNRER  185

TPK2      361  AKKVLGESMSISQFLDADIDCNGCVSKAEFVIYKLKKMDKITEKDINPIGFQFDKLDRTN  420
Chimera   282  AKKVLGESISISQFFAADIDNDGRLSLAEFAIYKLKQMEKITQEDFIQICNQFDKLDRTQ  341
KCO3      186  AKKVLGESISISQFFAADIDNDGRLSLAEFAIYKLKQMEKITQEDFIQICNQFDKLDRTQ  245

TPK2      421  SGRITLLDLLESSTKDLPTATSI             443
Chimera   342  SGRITLVDLTTATSV--------             356
KCO3      246  SGRITLVDLTTATSV--------             260
```

Supplemental Figure S2. Sequence alignment of KCO3-TPK2 chimera.
The amino acid sequences of TPK2 (cyan, top) and KCO3 (green, bottom) were aligned using Clustal Omega. The amino acid sequence from T192 to F287 of TPK2 was inserted into the region between C112 and G113 of KCO3 to construct the chimera (middle). The GYG (GFG) signature sequences of K^+ channels are underlined.

KCO3ΔM1

MPMTPSEFKNRLLFGSLPRSSSDPTDLQFTEPNVPPSLFSLPEHNDDTAT 50
DMAPDQETEQSVSKSIARQALALLVVYLSLGVLIYWLTLDSDNAYQTHPV 100
AVALYFFVVTFCGFLIVHFVVKIGWLDSFCFSVMMVTTVGFGDRAFNTWL 150
GTFLAAVWLLVSTLAVARAFLFLADARADKRNRERAKKVLGESISISQFF 200
AADIDNDGRLSLAEFAIYKLKQMEKITQEDFIQICNQFDKLDRTQSGRIT 250
LVDLTTATSV 260

KCO3M’

MPMTPSEFKNRLLFGSLPRSSSDPTDLQFTEPNVPPSLFSLPEHNDDTAT 50
DMAPDQETEQSVSKSIARQALALLVVYLSLGVLIYWLTLDSDNAYQTHPV 100
AVALYFFVVTFCGFLIVHFVVKIGWLDSFCFSVMMVTTVGFGDRAFNTWL 150
GTFLAAVWLLVSTLAVARAFLFLADARADKRNRERAKKVLGESISISQFF 200
AADIDNDGRLSLAEFAIYKLKQMEKITQEDFIQICNQFDKLDRTQSGRIT 250
LVDLTTATSV 260

Supplemental Figure S3. Amino acid sequences of KCO3ΔM1 (top) and KCO3M’ (bottom).

For each construct the entire sequence of KCO3 is shown with the deleted amino acid residues highlighted in black: KCO3ΔM1 lacks residues L71-A101 and KCO3M’lacks residues Y85-I116. The GYG (GFG) signature sequences of K+ channels are underlined.
